# Prolonged exercise shifts ventilatory parameters at the moderate-to-heavy intensity transition

**DOI:** 10.1007/s00421-023-05285-2

**Published:** 2023-07-27

**Authors:** Julian D. Stevenson, Andrew E. Kilding, Daniel J. Plews, Ed Maunder

**Affiliations:** Sports Performance Research Institute New Zealand, Auckland University of Technology, Auckland, New Zealand

**Keywords:** Cycling, Thresholds, Durability, Fatigue resistance, Exercise, Duration

## Abstract

**Purpose:**

To quantify the effects of prolonged cycling on the rate of ventilation (${\dot{\text{V}}}_{{\text{E}}}$), frequency of respiration (F_R_), and tidal volume (V_T_) associated with the moderate-to-heavy intensity transition.

**Methods:**

Fourteen endurance-trained cyclists and triathletes (one female) completed an assessment of the moderate-to-heavy intensity transition, determined as the first ventilatory threshold (VT_1_), before (PRE) and after (POST) two hours of moderate-intensity cycling. The power output, ${\dot{\text{V}}}_{{\text{E}}}$, F_R_, and V_T_ associated with VT_1_ were determined PRE and POST.

**Results:**

As previously reported, power output at VT_1_ significantly decreased by ~ 10% from PRE to POST. The ${\dot{\text{V}}}_{{\text{E}}}$ associated with VT_1_ was unchanged from PRE to POST (72 ± 12 vs. 69 ± 13 L^.^min^−1^, ∆ − 3 ± 5 L^.^min^−1^, ∆ − 4 ± 8%, *P* = 0.075), and relatively consistent (within-subject coefficient of variation, 5.4% [3.7, 8.0%]). The ${\dot{\text{V}}}_{{\text{E}}}$ associated with VT_1_ was produced with increased F_R_ (27.6 ± 5.8 vs. 31.9 ± 6.5 breaths^.^min^−1^, ∆ 4.3 ± 3.1 breaths^.^min^−1^, ∆ 16 ± 11%, *P* = 0.0002) and decreased V_T_ (2.62 ± 0.43 vs. 2.19 ± 0.36 L^.^breath^−1^, ∆ − 0.44 ± 0.22 L^.^breath^−1^, ∆ − 16 ± 7%, *P* = 0.0002) in POST.

**Conclusion:**

These data suggest prolonged exercise shifts ventilatory parameters at the moderate-to-heavy intensity transition, but ${\dot{\text{V}}}_{{\text{E}}}$ remains stable. Real-time monitoring of ${\dot{\text{V}}}_{{\text{E}}}$ may be a useful means of assessing proximity to the moderate-to-heavy intensity transition during prolonged exercise and is worthy of further research.

## Introduction

Power output at the boundaries between the moderate, heavy, and severe intensity domains are routinely used to assess performance capability, regulate training load and competition intensities, and to quantify adaptations to training (Burnley and Jones 2018; Jones et al. 2019; Maunder et al. 2021). However, we and others have observed that the power outputs observed at these intensity transitions decreases over time during prolonged exercise (Clark et al. 2018a, 2019a, b; Stevenson et al. 2022). This has implications for the application of physiological profiling data collected in well-rested athletes to prolonged training sessions (Maunder et al. 2021).

Identification of a physiological marker that changes over time during prolonged exercise in accordance with changes in the intensity domain transitions would be useful for within-session intensity regulation, and could result in a more precise calculation of training intensity distribution (Maunder et al. 2021). We previously observed that the classic upward drift in heart rate during prolonged cycling was proportionally greater than the downward drift in power output at the moderate-to-heavy intensity transition (Stevenson et al. 2022). This indicates that the heart rate observed at the moderate-to-heavy intensity transition during a physiological profiling assessment in a well-rested athlete may not provide useful information regarding the athlete’s proximity to the transition following multiple hours of exercise. Therefore, investigation of other markers of exercise intensity for this purpose are warranted.

Expired minute ventilation (${\dot{\text{V}}}_{{\text{E}}}$), and its underlying parameters respiratory frequency (F_R_) and tidal volume (V_T_), can be measured non-invasively by endurance athletes in real-time (Clarenbach et al. 2005; Witt et al. 2006; Nicolò et al. 2017b). These ventilatory parameters are highly-responsive to exercise intensity (Nicolò et al. 2017a, 2018). The ${\dot{\text{V}}}_{{\text{E}}}$ and F_R_ typically rise linearly with exercise intensity up to the respiratory compensation point, after which non-linear increases occur; whereas V_T_ typically plateaus at higher intensities (Nicolò et al. 2020). There is emerging suggestion that V_T_ may be regulated primarily by stimulation of central and peripheral chemoreceptors and skeletal muscle metaboreceptors by exercise-induced changes in CO_2_, pH, and skeletal muscle metabolites, whereas F_R_ may be primarily regulated by fast inputs such as group III/IV muscle afferents and central command (Tipton et al. 2017; Nicolò et al. 2020). Accordingly, monitoring changes in ventilatory parameters during exercise may have the potential to provide endurance athletes with useful information regarding their physiological status in real-time. This contention is further supported by the upward drift in ventilatory parameters that may be observed during prolonged, constant-work rate exercise (Phillips et al. 2016; Katagiri et al. 2023). If prolonged exercise-induced changes in these ventilatory parameters align with prolonged exercise-induced changes in the intensity domain transitions; that is, if one or more ventilatory parameters coincident with the intensity domain transitions remains constant over time during prolonged exercise, despite reductions in external work rates achieved at the transition, then monitoring ventilatory parameters during exercise may provide athletes with useful information regarding their real-time proximity to the intensity domain transitions.

Accordingly, the purpose of the present investigation was to quantify the effects of prolonged cycling on the ${\dot{\text{V}}}_{{\text{E}}}$, F_R_, and V_T_ associated with the moderate-to-heavy intensity transition. The data presented here were collected as part of a previously-published study (Stevenson et al. 2022).

## Methods

### Ethical approval

This study was performed in accordance with the standards of the Declaration of Helsinki, 2013. The Auckland University of Technology Ethics Committee approved all procedures (21/253), and all participants provided written informed consent prior to participation. This study was not registered in a database. Data associated with this study are available from the corresponding author upon reasonable request.

### Participants

Fourteen endurance-trained cyclists and triathletes took part in the present investigation (13 males, 1 female; age, 34 ± 10 y; height, 178.1 ± 5.6 cm; mass, 71.2 ± 6 kg; peak oxygen uptake [${\dot{\text{V}}}$O_2_peak], 59.9 ± 6.8 mL^.^kg^−1.^min^−1^; training volume, 9 ± 3 h^.^week^−1^). A priori sample size estimation indicated that a total sample size of 15 was required to detect a large magnitude (ES = 0.7) reduction in power output at the moderate-to-heavy intensity transition with 80% statistical power using the G*Power software package. A large magnitude effect size was used for this calculation based on previous studies showing the effect of prolonged exercise on the heavy-to-severe intensity transition (Clark et al. 2018b, 2019a, b). A one-tailed test was used as it seemed implausible that the moderate-to-heavy intensity power output would increase following acute prolonged exercise. One participant dropped out of the study. All participants were free of recent (< 3 months) musculoskeletal injury and chronic disease and habitually training > 5 h^.^week^−1^ in cycling-based endurance sports.

### Study design

The data presented here were collected as part of a previously-published study (Stevenson et al. 2022). Briefly, participants reported to the laboratory following an overnight fast on two occasions: (i) a characterisation trial for measurement of ${\dot{\text{V}}}$O_2_peak and initial estimation of the moderate-to-heavy intensity transition, (ii) a prolonged trial for measurement of the moderate-to-heavy intensity transition before and after two hours of cycling at 90% of the initial estimate of the moderate-to-heavy intensity transition. The first ventilatory threshold (VT_1_) was used as the marker of the moderate-to-heavy intensity transition.

### Characterisation trial

Participants initially reported to the laboratory for an incremental cycling test. Participants arrived after a 10-h overnight fast having refrained from vigorous exercise for 24 h and having ingested ~ 1 L of plain water ~ 2 h beforehand. Height and body mass was first measured. Cycling subsequently commenced on an electromagnetically-braked ergometer at 95 W, and the power output initially increased by 35 W every 3 min (Excalibur Sport, Lode BV, Groningen, NET). Expired gases were collected continuously using indirect calorimetry (TrueOne 2400, ParvoMedics, UT, USA). Once the respiratory exchange ratio exceeded 1.0 and clear signs of increased ${\dot{\text{V}}}_{{\text{E}}}$.${\dot{\text{V}}}$ O_2_^−1^ emerged, power output was increased by 35 W every minute until task failure. The ${\dot{\text{V}}}$O_2_peak was identified as the highest 15-s average ${\dot{\text{V}}}$O_2_, and VT_1_ was identified as the ${\dot{\text{V}}}$O_2_ at which a systematic rise in ${\dot{\text{V}}}_{{\text{E}}}$^.^${\dot{\text{V}}}$O_2_^−1^ occurred. This ${\dot{\text{V}}}$O_2_ was converted to a power output by linear fit of the power output vs. ${\dot{\text{V}}}$O_2_ relationship, using the last minute of ${\dot{\text{V}}}$O_2_ data from each 3-min stage.

### Prolonged trial

Participants arrived for the prolonged trial after a 10-h overnight fast, having refrained from vigorous exercise for 24 h, and having ingested ~ 1 L of plain water ~ 2 h beforehand. Following measurement of body mass, the experimental trial commenced on the same electromagnetically-braked ergometer as the initial assessment with a 5-min warm-up at 100 W, followed by a five-stage incremental assessment to determine the power output and heart rate at the moderate-to-heavy intensity transition (PRE). The incremental test began with 4-min at 50 W below the previously estimated VT_1_ power output, and power output increased by 25 W per increment. Expired gases were measured continuously during the incremental test (TrueOne 2400, ParvoMedics, UT, USA; Tickr, Wahoo Fitness, Atlanta, USA). Participants then cycled for 5 min at 100 W, and then at 90% of the previously estimated power output at VT_1_ for 2 h. Participants consumed plain water ad libitum. Following the two-hour constant work-rate phase, participants again cycled for 5 min at 100 W before repeating the five-step incremental exercise assessment (POST).

The moderate-to-heavy intensity transitions in PRE and POST were estimated using the VT_1_ method in accordance with the procedures described above for the initial assessment. The ${\dot{\text{V}}}$O_2_ at VT_1_ was converted to a power output by linear fit of the power output vs. ${\dot{\text{V}}}$O_2_ relationship, using the last minute of ${\dot{\text{V}}}$O_2_ data from each of the five 4-min stages. The VT_1_ was then matched to a corresponding ${\dot{\text{V}}}_{{\text{E}}}$, F_R_, and V_T_ value by linear fit of the relationship between these variables and power output using the last minute of data from each stage. The linear fit of individual ${\dot{\text{V}}}_{{\text{E}}}$ (R^2^ = 0.977 ± 0.015), F_R_ (R^2^ = 0.927 ± 0.061), and V_T_ (R^2^ = 0.829 ± 0.278) against power output curves were strong.

The validity of our VT_1_ data for estimating the moderate-to-heavy intensity transition is supported by its alignment with the blood lactate-derived LoglogLT estimate (within-subject coefficient or variation, ~ 6.9%) and lack of significant difference between VT_1_ and LoglogLT (*P* = 0.18), as reported in our previous publication related to this data collection (Stevenson et al. 2022). Specifically, we collected capillary blood lactate samples in the last 30-s of each stage, and the blood lactate concentration vs. power output relationship was used to quantify LoglogLT. The LoglogLT method models a blood lactate concentration vs. power output curve using two segments, and the intersection point of the two lines with the lowest residuals sum of squares is taken as the moderate-to-heavy intensity transition (Jamnick et al. 2018). Whilst no accepted gold-standard estimate of the moderate-to-heavy intensity transition exists, the alignment of these two separate estimates supports their validity.

### Statistical analysis

Data are presented as mean ± standard deviation (SD), unless otherwise stated. Normality of data distributions were assessed using the Shapiro–Wilk test. The effect of prolonged exercise on VT_1_, expressed as power output (previously reported (Stevenson et al. 2022)), ${\dot{\text{V}}}_{{\text{E}}}$, F_R_, and V_T_, was assessed using paired t-tests (or non-parametric equivalents). These analyses were performed in GraphPad Prism Version 9.3.1. The consistency of values between PRE and POST was assessed using within-subject coefficient of variation (CV) statistics calculated using the within-standard deviation method and Pearsons’s product-moment correlations, both expressed with 95% confidence intervals. These analyses were performed in R (version 4.4.0) with RStudio (version 1.1463). Significance was inferred when *P* ≤ 0.05.

## Results

**Fig. 1:**
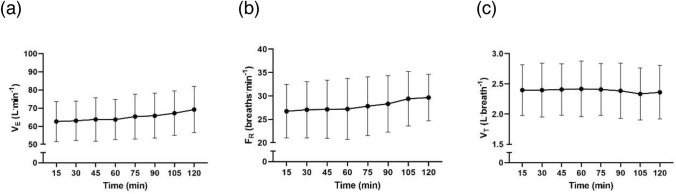
**a** Rate of ventilation (${\dot{\text{V}}}_{{\text{E}}}$), **b** frequency of respiration (F_R_), and **c** tidal volume (V_T_) during constant-work rate cycling between PRE and POST

**Fig. 2:**
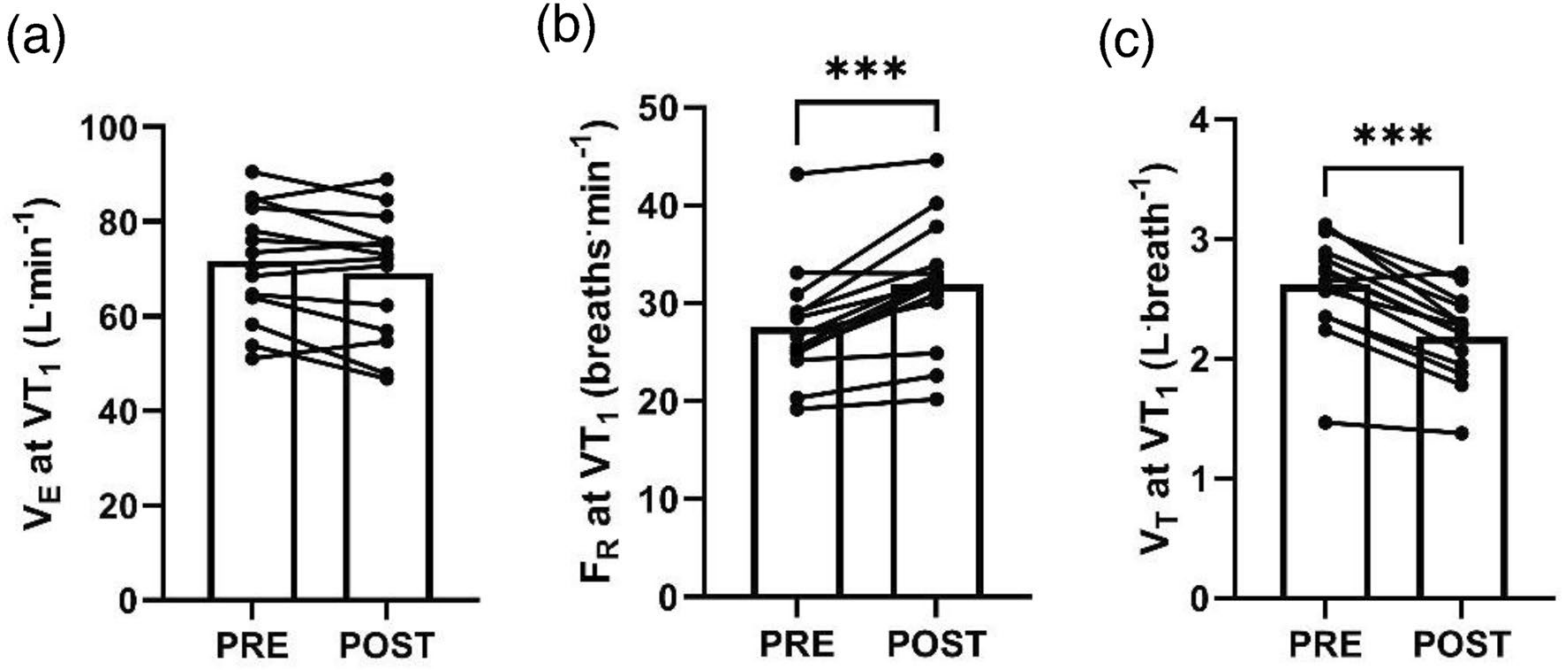
**a** Rate of ventilation (${\dot{\text{V}}}_{{\text{E}}}$), **b** frequency of respiration (F_R_), and (**c**) tidal volume (V_T_) at the first ventilatory threshold (VT_1_) before (PRE) and after (POST) prolonged cycling. *** denotes *P* ≤ 0.001

**Fig. 3:**
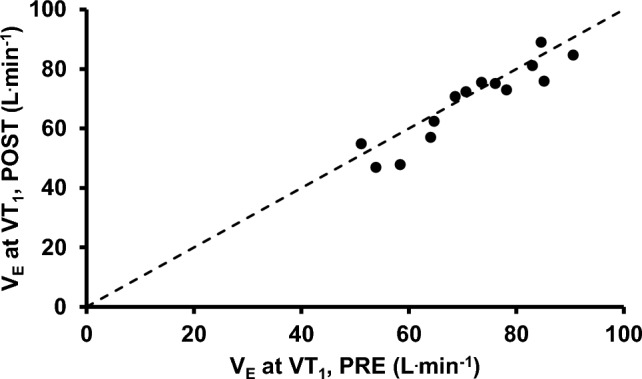
Rate of ventilation (${\dot{\text{V}}}_{{\text{E}}}$) at the first ventilatory threshold (VT_1_) before (PRE) and after (POST) prolonged cycling. The dashed line indicates x = y

**Fig. 4:**
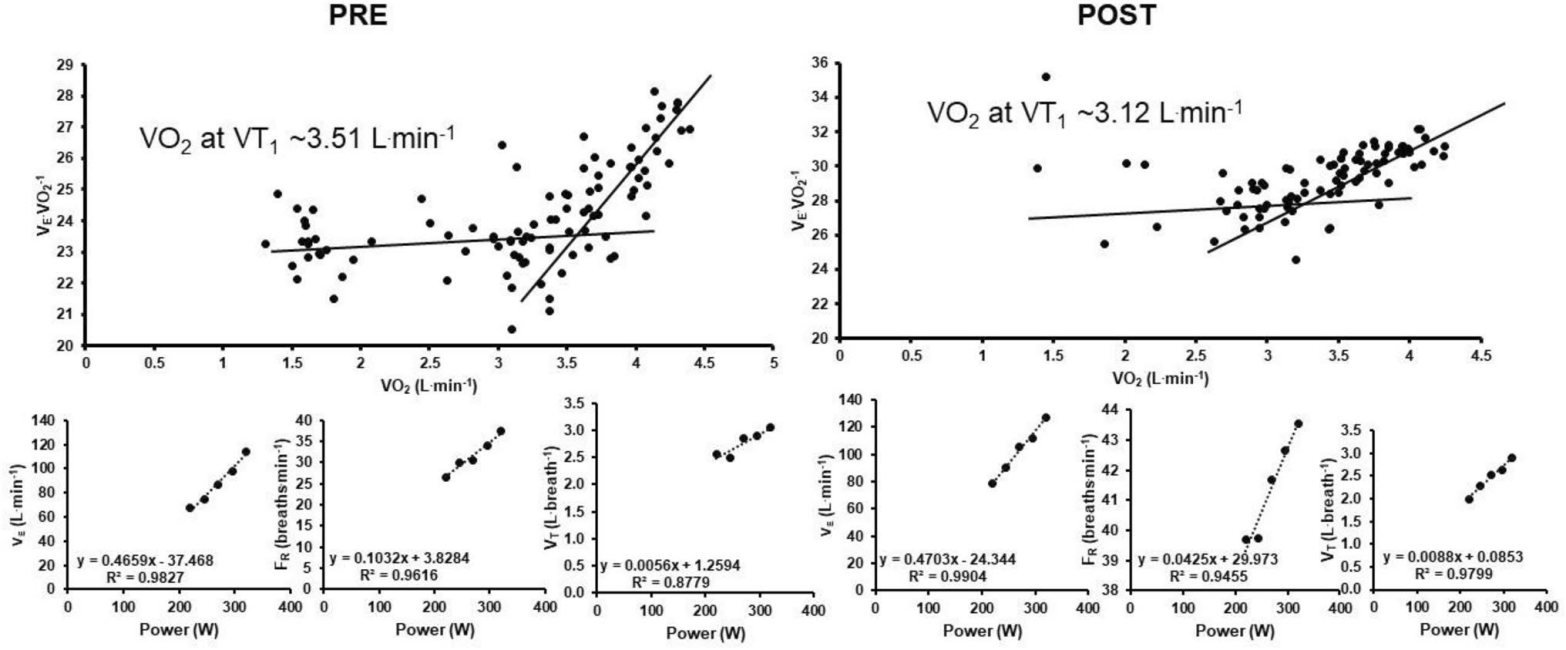
Data from a representative participant. Estimates of the rate of oxygen consumption (${\dot{\text{V}}}$O_2_) at the first ventilatory threshold (VT_1_) are shown for PRE and POST, as are the linear fittings of the rate of ventilation (${\dot{\text{V}}}_{{\text{E}}}$), frequency of respiration (F_R_), and tidal volume (V_T_) against power output

Power output at VT_1_ significantly decreased from PRE to POST (217 ± 42 W vs. 196 ± 42 W, ∆ − 21 ± 12 W, ∆ − 10 ± 6%, *P* < 0.001) (Stevenson et al. 2022). During the moderate-intensity, constant-work rate phase between PRE and POST, ${\dot{\text{V}}}_{{\text{E}}}$ (*P* = 0.065), F_R_ (*P* = 0.068), and V_T_ (*P* = 0.266) did not significantly change with time (Fig. 1). The ${\dot{\text{V}}}_{{\text{E}}}$ at VT_1_ was unchanged from PRE to POST (72 ± 12 vs. 69 ± 13 L^.^min^−1^, ∆ − 3 ± 5 L^.^min^−1^, ∆ − 4 ± 8%, *P* = 0.075, Fig. 2a), whereas F_R_ at VT_1_ increased (27.6 ± 5.8 vs. 31.9 ± 6.5 breaths^.^min^−1^, ∆ 4.3 ± 3.1 breaths^.^min^−1^, ∆ 16 ± 11%, *P* < 0.001, Fig. 2b) and V_T_ decreased (2.62 ± 0.43 vs. 2.19 ± 0.36 L^.^breath^−1^, ∆ − 0.44 ± 0.22 L^.^breath^−1^, ∆ − 16 ± 7%, *P* < 0.001, Fig. 2c) from PRE to POST. The within-subject CV for ${\dot{\text{V}}}_{{\text{E}}}$ at VT_1_ between PRE and POST was 5.4% (3.7, 8.0%), and the PRE and POST values were strongly associated (r = 0.928 [0.782, 0.977], *P* < 0.001) (Fig. 3). Data from a representative participant is shown in Fig. 4.

## Discussion

The aim of the present investigation was to assess the effect of prolonged exercise on the ${\dot{\text{V}}}_{{\text{E}}}$, and its underlying parameters F_R_ and V_T_, associated with the moderate-to-heavy intensity transition. Our primary observations were, concomitant with a reduction in the power output associated with the moderate-to-heavy intensity transition: (i) the ${\dot{\text{V}}}_{{\text{E}}}$ associated with the transition was unchanged, whilst (ii) the associated F_R_ increased and (iii) V_T_ decreased. These data suggest that real-time monitoring of ${\dot{\text{V}}}_{{\text{E}}}$ may be a useful means of assessing proximity to the moderate-to-heavy intensity transition during prolonged exercise.

We previously observed that the heart rate associated with the moderate-to-heavy intensity transition increased following prolonged cycling (Stevenson et al. 2022). These data indicated that heart rate thresholds, measured in traditional, well-rested athlete physiological profiling assessments, may not readily translate to prolonged exercise. Specifically, adherence to heart rate zones derived from well-rested athlete physiological profiling assessments may risk undertraining an athlete during prolonged exercise as the heart rate associated with the intensity transition drifts upwards over time. Here we present evidence that ${\dot{\text{V}}}_{{\text{E}}}$ may be an alternative physiological metric that can be used to provide information regarding the proximity to the moderate-to-heavy intensity transition during exercise, as the ${\dot{\text{V}}}_{{\text{E}}}$ associated with the transition was unchanged following prolonged cycling. Whilst not statistically significant (*P* = 0.075), our data does show a numeric decrease in the ${\dot{\text{V}}}_{{\text{E}}}$ associated with VT_1_ from PRE to POST (Fig. 2a). However, even if this effect was statistically significant, we consider this numeric reduction to be practically insignificant in terms of magnitude (∆ − 4 ± 8%), given the reported day-to-day variation associated with gas exchange-derived estimates of ${\dot{\text{V}}}_{{\text{E}}}$ (~ 4–7%) (Carter and Jeukendrup 2002). Therefore, estimates of the ${\dot{\text{V}}}_{{\text{E}}}$ associated with the moderate-to-heavy intensity transition during traditional physiological profiling assessments may translate effectively to prolonged exercise, and therefore to within-session intensity regulation and calculation of training intensity distribution. That is, if an athlete’s moderate-to-heavy intensity transition power output in a well-rested physiological assessment is determined as 215 W with a concomitant ${\dot{\text{V}}}_{{\text{E}}}$ of 70 L^.^min^−1^, our data suggests their moderate-to-heavy intensity transition power output after prolonged exercise may fall, but the ${\dot{\text{V}}}_{{\text{E}}}$ at the moderate-to-heavy transition would remain constant at 70 L^.^min^−1^. Therefore, if this athlete intends to undertake a prolonged training session in the moderate domain, they could guide their effort according to keeping ${\dot{\text{V}}}_{{\text{E}}}$ below 70 L^.^min^−1^, although the values used in practice should acknowledge the day-to-day variation in exercise ${\dot{\text{V}}}_{{\text{E}}}$.

Our observation that ${\dot{\text{V}}}_{{\text{E}}}$ is tightly linked with physiologically-based intensity domain transitions is highly-plausible, given the physiological stresses associated with increased exercise intensity regulate ${\dot{\text{V}}}_{{\text{E}}}$ (Tipton et al. 2017; Nicolò et al. 2020). For example, transition from the moderate to heavy intensity domain sees perturbations in muscle metabolic homeostasis, including increased lactate and H^+^ accumulation and depletion of PCr stores (Black et al. 2017). Disturbed muscle metabolic homeostasis drives hyperpnoea via stimulation of muscle metaboreceptors (Piepoli et al. 1995; Stickland et al. 2013). The increased F_R_ and decreased V_T_ used to produce the constant rate of ${\dot{\text{V}}}_{{\text{E}}}$ at the moderate-to-heavy intensity transition in POST vs. PRE might reflect fatigue in the respiratory musculature, and therefore a shift in the most efficient ventilatory pattern to produce a given rate of ${\dot{\text{V}}}_{{\text{E}}}$, following prolonged exercise. This would align with the so-called ‘principle of minimal effort’ (Otis et al. 1950; Mead 1960), but requires examination in specific work to be confirmed.

The translation of these data to practical settings requires wearable technology that can accurately and reliably measure ventilatory parameters during training. Several technologies exist that allow for accurate estimation of F_R_ in real-time during exercise (Nicolò et al. 2017b), including through sensors embedded in straps or clothes sensitive to thoracic or abdominal strain (Hailstone and Kilding 2011; Kim et al. 2013; Liu et al. 2013; Villar et al. 2015), or ventilation-induced changes in the electrocardiogram or photoplethysmography signals (Bailón et al. 2006; Meredith et al. 2012; Schumann et al. 2016). Respiratory-inductive plethysmography has been used to estimate F_R_, V_T_ and ${\dot{\text{V}}}_{{\text{E}}}$, with some success (Clarenbach et al. 2005; Witt et al. 2006). Therefore, development of technologies that can be used by endurance athletes to estimate real-time ${\dot{\text{V}}}_{{\text{E}}}$ during exercise, with data integrated onto bicycle computers and/or smart watches such that it can be viewed in real-time, may allow for improved within-session intensity regulation when coupled with quantification of the ${\dot{\text{V}}}_{{\text{E}}}$ associated with the moderate-to-heavy intensity transition during routine physiological profiling assessments. However, as indicated above, intensity-related decisions using measurements of ${\dot{\text{V}}}_{{\text{E}}}$ in practice would need to acknowledge the day-to-day variation in exercise ${\dot{\text{V}}}_{{\text{E}}}$ measured using said device.

Additionally, translation of these data to applied settings requires further research to address the limitations of our study. For example, our observations are specific to our exercise protocol; that is, a submaximal incremental test followed by two hours of initially moderate-intensity exercise. It is possible that the main effects observed here do not translate to intermittent or higher-intensity exercise protocols, or longer exercise durations. Similarly, our data were collected during exercise in a fasted state without feeding during exercise, and (primarily) in male athletes. Thus, further research is warranted to determine if ${\dot{\text{V}}}_{{\text{E}}}$ at the moderate-to-heavy intensity transition remains constant over time during prolonged exercise across a broader range of ecologically-valid exercise scenarios.

In summary, the data presented here indicate that real-time monitoring of ${\dot{\text{V}}}_{{\text{E}}}$ during prolonged exercise might provide a useful means of assessing proximity to the moderate-to-heavy intensity transition. This would require prior assessment of the ${\dot{\text{V}}}_{{\text{E}}}$ associated with the moderate-to-heavy intensity transition during routine physiological profiling assessments, and technologies that allow accurate, real-time monitoring of ${\dot{\text{V}}}_{{\text{E}}}$ during exercise.

## Data Availability

Data is available from the corresponding author upon reasonable request.

## Abbreviations

F_R_: Frequency of respiration
HR: Heart rate
${\dot{\text{V}}\text{CO}}_{{2}}$: Rate of carbon dioxide production
${\dot{\text{V}}}_{{\text{E}}}$: Expired minute ventilation
${\dot{\text{V}}\text{O}}_{{2}}$: Rate of oxygen consumption
${\dot{\text{V}}\text{O}}_{{2}} {\text{peak}}$: Peak rate of oxygen consumption
V_T_: Tidal volume
VT_1_: First ventilatory threshold

## References

[CR1] Bailón R, Sörnmo L, Laguna P (2006). A robust method for ECG-based estimation of the respiratory frequency during stress testing. IEEE Transl Biomed Eng.

[CR2] Black MI, Jones AM, Blackwell JR (2017). Muscle metabolic and neuromuscular determinants of fatigue during cycling in different exercise intensity domains. J Appl Physiol.

[CR3] Burnley M, Jones AM (2018). Power–duration relationship: Physiology, fatigue, and the limits of human performance. Eur J Sport Sci.

[CR4] Carter J, Jeukendrup AE (2002). Validity and reliability of three commercially available breath-by-breath respiratory systems. Eur J Appl Physiol.

[CR5] Clarenbach CF, Oliver S, Thomas B (2005). Monitoring of ventilation during exercise by a portable respiratory inductive plethysmograph. Chest.

[CR6] Clark IE, Vanhatalo A, Bailey SJ (2018). Effects of two hours of heavy-intensity exercise on the power-duration relationship. Med Sci Sports Exerc.

[CR7] Clark IE, Vanhatalo A, Bailey SJ (2018). Effects of two hours of heavy-intensity exercise on the power–duration relationship. Med Sci Sports Exerc.

[CR8] Clark IE, Vanhatalo A, Thompson C (2019). Dynamics of the power-duration relationship during prolonged endurance exercise and influence of carbohydrate ingestion. J Appl Physiol.

[CR9] Clark IE, Vanhatalo A, Thompson C (2019). Changes in the power-duration relationship following prolonged exercise: estimation using conventional and all-out protocols and relationship with muscle glycogen. Am J Physiol - Regul Integr Comp Physiol.

[CR10] Hailstone J, Kilding AE (2011). Reliability and validity of the Zephyr™ BioHarness™ to measure respiratory rate responses to exercuse. Meas Physiocal Educ Exerc Sci.

[CR11] Jamnick NA, Botella J, Pyne DB, Bishop DJ (2018). Manipulating graded exercise test variables affects the validity of the lactate threshold and VO2peak. PLoS ONE.

[CR12] Jones AM, Burnley M, Black MI (2019). The maximal metabolic steady state: redefining the ‘gold standard’. Physiol Rep.

[CR13] Katagiri A, Fujii N, Dobashi K (2023). Sodium bicarbonate reduces ventilation without altering core temperature threshold or sensitivity of hyperthermia-induced hyperventilation in exercising humans. Am J Physiol - Regul Integr Comp Physiol.

[CR14] Kim JH, Roberge R, Powell JB (2013). Measurement accuracy of heart rate and respiratory rate during graded exercise and sustained exercise in the heat using the Zephyr BioHarness. Int J Sports Med.

[CR15] Liu Y, Zhu SH, Wang GH (2013). Validity and reliability of multiparameter physiological measurements recorded by the equivital lifemonitor during activities of various intensities. J Occup Environ Hyg.

[CR16] Maunder E, Seiler S, Mildenhall MJ (2021). The importance of ‘durability’ in the physiological profiling of endurance athletes. Sports Med.

[CR17] Mead J (1960). Control of respiratory frequency. J Appl Physiol.

[CR18] Meredith DJ, Clifton D, Charlton P (2012). Photoplethysmographic derivation of respiratory rate: a review of relevant physiology. J Med Eng Technol.

[CR19] Nicolò A, Marcora SM, Bazzucchi I, Sacchetti M (2017). Differential control of respiratory frequency and tidal volume during high-intensity interval training. Exp Physiol.

[CR20] Nicolò A, Massaroni C, Passfield L (2017). Respiratory frequency during exercise: The neglected physiological measure. Front Physiol.

[CR21] Nicolò A, Girardi M, Bazzucchi I (2018). Respiratory frequency and tidal volume during exercise: differential control and unbalanced interdependence. Physiol Rep.

[CR22] Nicolò A, Marcora SM, Sacchetti M (2020). Time to reconsider how ventilation is regulated above the respiratory compensation point during incremental exercise. J Appl Physiol.

[CR23] Otis AB, Fenn WO, Rahn N (1950). Mechanics of breathing in man. J Appl Physiol.

[CR24] Phillips DB, Stickland MK, Petersen SR (2016). Ventilatory responses to prolonged exercise with heavy load carriage. Eur J Appl Physiol.

[CR25] Piepoli M, Clark AL, Coats AJ (1995). Muscle metaboreceptors in hemodynamic, autonomic, and ventilatory responses to exercise in men. Am J Physiol - Hear Circ Physiol.

[CR26] Schumann A, Schmifdt M, Herbsleb M (2016). Deriving respiration from high resolution 12-channel-ECG during cycling exercise. Curr Dir Biomed Eng.

[CR27] Stevenson JD, Kilding AE, Plews DJ, Maunder E (2022). Prolonged cycling reduces power output at the moderate-to-heavy intensity transition. Eur J Appl Physiol.

[CR28] Stickland MK, Lindinger MI, Olfert M (2013). Pulmonary gas exchange and acid-base balance during exercise. Compr Physiol.

[CR29] Tipton MJ, Harper A, Paton JFR, Costello JT (2017). The human ventilatory response to stress: rate or depth?. J Physiol.

[CR30] Villar R, Beltrame T, Hughson RL (2015). Validation of the Hexoskin wearable vest during lying, sitting, standing, and walking activities. Appl Physiol Nutr Metab.

[CR31] Witt JD, Fisher JRKO, Guenette JA (2006). Measurement of exercise ventilation by a portable respiratory inductive plethysmograph. Respir Physiol Neurobiol.

